# Protoplasm

**Published:** 1881-04

**Authors:** S. O. Gleason


### PROTOPLASM.

S. O. GLEASON, M. D.

This term is derived from the Greek, *Protos*, first, *plasso*, to form. It is a colorless, smooth or granular viscid substance, homogeneous in character. It is readily detected under the microscope by the ease with which it combines with coloring matter, such as aniline and carmine. It coagulates in alcohol, in mineral acids and by heat.

This substance resembles other albumoids and consists of carbon, oxygen, hydrogen, nitrogen and a small proportion of sulphur. It has the power to absorb water in various quantities which makes it nearly liquid, while at other times it becomes firm and leathery.

Its more permanent qualities are excitability and contractility. Protoplasm is called the "physical basis of life," the original substance from which all living beings are developed and is present in every phase of life. All that is comprehended under the term life, whether in the growth of plants or animals, in the flight of birds, or train of human thought, is supposed to be caused by organs which consist of protoplasm, or have been developed out of it.

It is present wherever there is nutrition or propogation,. motion or sensation. There are certain protozoans—called *monera*, the entire body of which, with all its capabilities is made up and consists solely of protoplasm. They are the simplest organizations with which we have any knowledge and the most minute and structureless of living beings that we can conceive as capable of existing. Their entire body is but a single formless mass or rather lump of protoplasm, with no combination of parts; yet, they perform all the functions which in their oneness, constitute the most highly organized animals and plants. They illustrate in their simple existence, the varied phenomena of life. Such as motion, sensation, nutrition and propogation. By studying these simple *monera*, we obtain a very clear and definite idea of the nature and importance of this living substance, called protoplasm.

These monera live in both fresh and salt water. As a rule they are invisible to the naked eye—while some of them are as large as the head of a pin. They are among the things that are of intense interest to the microscopist. When at rest the monad has a spherical shape. The surface of the body may be smooth—or very delicate threads may radiate from it in every direction. These thread-like extensions of its substance are not permanent organs. They come and go—they vary every moment in number, size and form. For this season, they are pseudopodia or false feet. Thus, the functions of the higher animals are performed by these simple means; for, by shortening or elongating these finger- or thread-like prolongations, they drag their bodies after them. If any point of the body or any filament be touched with the point of a needle—or any chemical substance, or current of electricity, the threads are drawn in and the body contracts and assumes a ball like shape. These filamentous prolongations perform the function of food providers. The pseudopodia when extended, come in contact with infusoria or other food particles, envelope them in their substance and convey them into the interior of the body, where the nutrition is absorbed and then the remainder is ejected as useless. The variations in the *monera* consist in many kinds of pseudopodia and in the many modes of reproduction. Some divide into halves; others put out small buds which in time separate from the parent; others still burst into numerous small round bodies each of which begins a separate life, until it reaches the size of its ancestor.

The single, simple cell is able to live as an independent organism. Many of the lowest plants and animals retain for life all the characteristics of a simple cell. The most primitive unicellar organism is the *amœba*.

The *monera* is supposed to have no germ, while the Amœba, as other cells, has this germ, an organ of propogation and heredity, while the other functions, alimentation, motion and sensation are performed by the protoplasmic body substance.

Thus, cells reproduce themselves by a division of the germ, around each half of which protoplasm gathers, until the main body separates into two distinct cells, each of which grows and subdivides an indefinite number of times.

Cells are elementary organisms, minute forms of life, which may live independently, or in higher forms, may combine in multitudes to form a community. So, it comes to pass that the various forms of life by differentiation of cells [brought about by the different chemical arrangement of the granular structure of the protoplasm] are evolved.

